# Do Self-Perceptions of Emotional Intelligence Predict Health-Related Quality of Life? A Case Study in Hospital Managers in Greece

**DOI:** 10.5539/gjhs.v7n1p210

**Published:** 2014-08-31

**Authors:** George Gourzoulidis, Nick Kontodimopoulos, Catherine Kastanioti, Thaleia Bellali, Konstantinos Goumas, Dikaios Voudigaris, Nikolaos Polyzos

**Affiliations:** 1 Department of Health and Welfare Unit Management, Higher Technological Educational Institute of Peloponnese, Peloponnese, Greece; 2 Hellenic Open University, Patra, Greece; 3 Faculty of Nursing, Alexandreio Technological Educational Institute of Thessaloniki, Thessaloniki, Greece; 4 Department of Social Management, Democritus University of Thrace, Komotini, Greece

**Keywords:** Quality of Life, emotional intelligence, hospital managers

## Abstract

The aim of this study was to examine HRQoL outcome and EI of managers of Health Organizations. We collected data from 120 general managers of Greek public hospitals who completed the Assessing Emotions Scale (AES) and the SF-36 Health Survey.

The results showed that male managers generally exhibited better HRQoL and slightly worse EI than females, although differences were not significant throughout. The three EI factors of the AES addressing appraisal, optimism/regulation and utilization of emotions correlated from 0.18 to 0.39 with sub-dimensions of HRQoL mostly related to mental -rather than physical- aspects of health, and were also significant predictors of HRQoL.

There was a noteworthy gender difference in the manner in which EI predicted HRQoL and this suggests more testing. Overall, this study might enrich the potential for EI studies in Greece as well as to contribute to the international literature.

## 1. Introduction

Over the past two decades there has been an increasing number of studies, from researchers and practitioners across disciplines, dealing with the concept of emotional intelligence (EI). However, despite its relatively short history, EI has been shown to be useful in understanding a wide range of human functions (Tsaousis & Nikolaou, 2005).

Trait EI (or trait emotional self-efficacy) concerns emotion-related dispositions and self-perceptions measured via self-report, whereas EI ability (or cognitive emotional ability) concerns emotion-related cognitive abilities measured via performance-based tests (Petrides et al., 2007). Empirical findings have revealed low correlations between ability and trait EI (O’Connor & Little, 2003; Warwick & Nettelbeck, 2004).

Trait EI is concerned with traits or behaviors such as empathy, assertiveness and optimism embedded in the personality and is measured via validated self-report inventories of typical behavior. There is a consensus that higher trait EI is associated with better interpersonal relationships (Mayer et al., 1999), academic achievement (Parker et al., 2004; Van Der Zee et al., 2002) and coping (Salovey et al., 2002). Trait EI is also considered a fundamental asset for primary leadership and managerial competency in the workplace (Freshman & Rubino, 2002). Managers with higher emotional intelligence skills/competencies may be more successful than their less emotionally intelligent counterparts (Caruso & Salovey, 2004; George, 2000), although this assertion may be weakened by the general lack of evidence of how trait EI is related to managerial outcomes (Murphy, 2006). For health care managers, the importance of trait EI as a core competency has been defined as a set of intrapersonal and interpersonal skills in self-awareness, self-regulation, self-motivation, social awareness, and social skills and has been recognized for over a decade (Freshman & Rubino, 2002).

The dimensions of Health-related quality of life (HRQoL) are a multidimensional concept that includes physical, mental and social well being and have been accepted as an important outcome measure of healthcare for a long time (Stewart & Ware, 1992). Significant relationships have been recorded between trait EI and HRQoL, and a review of recent studies showed that better mental, physical and psychosomatic health was associated with higher trait EI (Schutte et al., 2007). According to results from studies that have investigated the relationship between trait EI and health EI was measured by the Assessing Emotions Scale (AES) and health, measured with various instruments, higher trait EI was expected to be associated mostly with better mental health, (Austin et al., 2005; Saklofske et al., 2003; Schutte et al., 1998) and to some extent with better psychosomatic health (Brown & Schutte, 2006). The literature lacks studies assessing trait EI in health care professionals and hospital managers in particular. Furthermore, we are also unaware of studies addressing the relationship between trait EI and HRQoL in this group of professionals. In light of these arguments, the objective of this study was to determine the association between self-perceptions of trait EI and HRQoL in a large group of NHS hospital managers in Greece.

## 2. Methods

### 2.1 Sample and Data Collection

The data were collected in December 2011 during a meeting between the leadership of the Ministry of Health (MoH) and managers (CEOs) and deputy managers (in hospitals with +400 beds) of the 131 hospitals in the Greek National Health System. Overall, 120 managers, out of 152 initially approached (and out of 175 overall), agreed to participate (78.9% response rate) and self-completed a survey including the AES, the SF-36 and socio-demographic and work-related questions. The mean completion time was about 20 minutes. The MoH- through the Secretary General- granted ethical approval for the study and all participants provided informed consent.

### 2.2 Instruments

The AES investigates the aspects of EI and is based on the model of Salovey and Mayer (1999), in which the three main constructs are: i) appraisal and expression of emotion, ii) regulation of emotion, and iii) utilization of emotions in solving problems. It is comprised of 33 items, three of which (#5, #28 and #33) are reverse-scored. Responses are given on a 5-point Likert scale, where 1=*strongly disagree* and 5=*strongly agree*, and the total score is derived by summing up item responses. The 33 items have been identified as belonging to a single global EI factor that has demonstrated high internal consistency (Cronbach’s alpha=0.90) and has been validated by confirmation of correlations with theoretically related constructs (e.g. alexithimia, pessimism, depression), and between-group differences (e.g. therapists, prisoners, clients in a substance abuse program). Later studies having examined the AES’s structure suggested multifactorial solutions. The original one-, as well as the three-, four- and six-factor proposed solutions were tested with confirmatory factor analysis (Kun et al., 2010) and the three-factor structure (Austin et al., 2004) was shown to be the most plausible. The factors were described by the labels *“appraisal of* *emotions”*, *“optimism and regulation of emotions”* and *“intrapersonal and interpersonal* *utilization of emotions”*. In the present study we used the three-factor model and the Greek version of the AES was developed by translation and back-translation, during which, all inconsistencies were resolved.

HRQoL was measured with the SF-36 Health Survey (Ware & Sherboune, 1992) comprising eight dimensions: physical functioning, role physical, bodily pain, general health, vitality, social functioning, role emotional and mental health. Each dimension is scored on a 0-100 scale with 0 and 100 corresponding to worst and best health status respectively (Ware et al., 1993). The instrument was translated into Greek and its reliability and validity were established in a representative sample of adults, in which it showed high internal consistency reliability, convergent and discriminative validity and discrimination between groups of respondents in the expected manner (known-groups’ validity) on the basis of gender, age and socio-economic status (Papa et al., 2005).

### 2.3 Analysis

Although examining the psychometric properties of the AES was not a direct objective of this study, some evidence was required to increase the confidence level of the results. Scale internal consistency reliability was assessed via Cronbach’s alpha. The hypothesized three-factor structure of the AES was assessed by examining item-scale correlations. Item internal consistency is substantial when correlation between an item and its hypothesized scale is > 0.40, and item discriminant validity is successful when correlation between an item and its own scale is significantly higher (> 2 standard errors) than with other scales (Ware & Gandek, 1998). The AES factor scores were computed by summing the responses to the relevant items and expressed on a 1-5 scale, whereas the SF-36 scale scores were expressed on the standard 0-100 scale, with higher scores reflecting both better trait EI and HRQoL. Scores were also computed by gender as women consistently show higher trait EI, (Mayer et al., 1999; Schutte et al., 1998; Van Rooy et al., 2005) but lower HRQoL (Apolone & Mosconi, 1998; Papa et al., 2009). SF-36 scale scores were compared to the Greek norms (Papa et al., 2005) and differences were examined with independent samples t-test and non-parametric Mann-Whitney test. Inter-correlations among trait EI and HRQoL components were examined. To identify how trait EI predicted HRQoL, multiple linear regressions were performed on the SF-36 scales using stepwise variable inclusion and the trait EI components as predicting variables. All analyses were performed with SPSS version 17.0.

## 3. Results

The participants were 80 male and 40 female general managers of Greek National Health System hospitals ranging in age from 31 to 70 years, (mean = 51.3, standard deviation = 8.7 years 78.3% of the participants were married and 79.7% had children). All participants were higher education graduates, 45.8% had a M.Sc. and 31.6% and/or a Ph.D. Furthermore, 39.8% of the sample reported having received education in a management-related discipline. Their previous experience in the Greek National Health System ranged from 1 to 15 years, and the hospitals they managed ranged in size from 30 to 1000 beds (mean=331.3, median=252.0).

Cronbach’s alpha for the single-factor (i.e. 33-item) AES scale was 0.89, and practically identical to the 0.90 value reported by Schutte et al. (1998), Internal reliabilities for the three factors were 0.74, 0.65 and 0.77 (Table 1), again comparable to the values (0.71, 0.72 and 0.73) reported in the validation study of the three-factor solution (Austin et al., 2004). All correlations between items and their hypothetical scales exceeded the 0.40 criterion, thus supporting convergent construct validity. Discriminant validity was implied by stronger correlations between most items and their scale than with the other scales. Most of the unsuccessful discriminate validity tests concerned items of the second factor with the lowest internal consistency. It is worth mentioning that the three factors explained 95.8% of the variance in the overall single factor, implying a negligible loss of information by using only 24 of the 33 items to construct these factors.

**Table 1:** AES Internal consistency and item-scale correlations

Item	Description	Cronbach’s alpha		Item-scale correlations		

		Scale	Item deleted	Factor 1	Factor 2	Factor 3
*Factor 1: Appraisal of Emotions*						
5	Hard to understand nonverbal messages of others	0.738	0.717	0.642***	0.309***	0.051
15	I am aware of nonverbal messages I send others		0.689	0.683***	0.517***	0.302**
18	By looking at facial expressions, I recognize emotions people are experiencing		0.715	0.563***	0.545***	0.398***
25	I am aware of nonverbal messages other people send		0.677	0.729***	0.491***	0.168
29	I know what other people are feeling by looking at them		0.698	0.654***	0.313***	0.226*
32	I can tell how people are feeling by listening to the tone of their voice		0.713	0.604***	0.444***	0.402***
33	Difficult to understand why people feel the way they do		0.739	0.560***	0.316***	0.122
*Factor 2: Optimism and Regulation of Emotions*						
3	I expect to do well on most things I try	0.645	0.587	0.418***	0.605***	0.434***
10	I expect good things to happen		0.567	0.394***	0.666***	0.502***
12	When I experience a positive emotion, I know how to make it last		0.604	0.266**	0.591***	0.487***
14	I seek out activities that make me happy		0.575	0.363***	0.653***	0.529***
19	I know why my emotions change		0.604	0.428***	0.570***	0.286**
21	I have control over my emotions		0.636	0.294**	0.474***	0.071
28	When faced with a challenge, I give up because I believe I will fail		0.676	0.433***	0.439***	0.173
*Factor 3: Intrapersonal and Interpersonal Utilization of Emotions*						
2	When faced with obstacles, I remember times I overcame similar obstacles	0.769	0.741	0.218*	0.496***	0.622***
6	Major events of my life have led me to re-evaluate what is important		0.737	0.193*	0.357***	0.652***
7	When my mood changes, I see new possibilities		0.758	0.004	0.157	0.591***
17	When in a positive mood, solving problems is easy		0.752	0.099	0.289**	0.568***
20	When in a positive mood, I can come up with new ideas		0.736	0.135	0.356***	0.654***
23	I motivate myself by imagining a good outcome to my tasks		0.753	0.163	0.340***	0.549***
24	I compliment others when they have done something well.		0.766	0.398***	0.364***	0.421***
27	When I feel a change in emotions, I tend to come up with new ideas		0.762	0.266**	0.364***	0.527***
30	I help other people feel better when they are down		0.762	0.441***	0.428***	0.456***
31	I use good moods to help myself keep trying in the face of obstacles		0.730	0.290**	0.522***	0.697***

* *P*<0.05

** *P*<0.01

*** *P*<0.001.

Women managers scored higher on all three EI factors, however a significant difference was observed only for *appraisal of emotions* (*p*<0.01) (Table 2). Men, on the other hand, reported better HRQoL on all SF-36 scales and significant differences were recorded for *social* *functioning* (*p*<0.01) and *role emotional* (*p*<0.05). SF-36 scores were compared to the Greek norms and showed better health status (*p*<0.001 throughout all SF-36 scales and *p*<0.05 for *social functioning*) for the sample of hospital managers.

**Table 2:** AES and SF-36 scale means (SD) by gender and HRQOL comparisons with the Greek general population

AES factors	Males (N=80)	Females (N=40)	*P*-value	Study Sample (N=120)	Greek Norms (N=1007)	*P*-value
Appraisal of emotions	3.87 (0.53)	4.17 (0.50)	0.006			
ism/positivity	4.18 (0.42)	4.24 (0.52)	0.564			
Regulating/using emotions	4.15 (0.44)	4.29 (0.59)	0.176			
SF-36 scales						
Physical Functioning	95.0 (6.0)	90.1 (15.4)	0.420	93.5 (9.96)	79.5 (26.3)	<0.001
Role Physical	95.9 (12.3)	88.2 (27.7)	0.196	93.6 (18.5)	78.6 (38.7)	<0.001
Bodily Pain	93.0 (12.5)	87.1 (19.9)	0.128	90.9 (15.5)	72.4 (31.9)	<0.001
General Health	78.1 (12.3)	77.2 (15.8)	0.895	77.8 (13.4)	66.7 (23.8)	<0.001
Vitality	81.1 (11.2)	74.3 (22.5)	0.598	79.1 (15.5)	66.0 (22.5)	<0.001
Social Functioning	89.9 (16.8)	78.1 (26.8)	0.010	86.4 (21.0)	81.3 (28.7)	0.019
Role Emotional	95.8 (16.3)	86.1 (28.0)	0.011	92.9 (20.8)	81.2 (36.6)	<0.001
Mental Health	81.9 (11.6)	73.3 (21.4)	0.074	79.5 (15.7)	68.2 (21.2)	<0.001

Also, the strong relationships between components of trait EI and mental health were expected and the weak associations with physical health were confirmed (Table 3).

**Table 3:** Correlations of emotional intelligence and SF-36 scales

SF-36 scales	Emotional intelligence subscales		

	Appraisal of emotions	Optimism/regulation	Utilization of emotions
Physical Functioning	0.12	0.18	-.0.03
Role Physical	0.17	0.11	-0.14
Bodily Pain	0.01	0.04	-0.03
General Health	0.39***	0.39***	0.23*
Vitality	0.35***	0.29**	0.08
Social Functioning	0.17	0.09	-0.06
Role Emotional	0.02	0.01	-0.18*
Mental Health	0.28**	0.33***	0.04

* *P*<0.05

** *P*<0.01

*** *P*<0.001.

In the overall sample, *optimism/regulation of emotions* and *utilization of emotions* were significant predictors of four and five health SF-36 scales respectively, whereas *appraisal of emotions* did not predict any HRQoL dimension (Table 4). Furthermore, *physical functioning* and *bodily pain* were not predicted by any EI factor. The highest portion of explained variance was in the *general health* and *mental health* scales (13.6% and 12.0% respectively). The predictive performance of trait EI on HRQoL differed by gender. For men, *utilizing* *emotions* did not predict HRQoL, whereas *appraisal* and *optimism/regulation* were significant predictors for four and two SF-36 scales respectively. Significant variance was explained for *general health* (24.1%) and *mental health* (15.1%). Contrarily in females, *appraisal of* *emotions* was not a significant predictor of HRQoL, assimilating the result for the overall sample, and HRQoL was predicted primarily by *utilizing emotions* (five SF-36 scales) and *optimism/regulation* (one scale). Noteworthy variance was explained for *role emotional* (16.8%) and *role physical* (13.6%).

**Table 4:** Multivariate analyses [Β coefficient (p-sig.)]

Predictors		SF-36 scales							
	
			PF	RP	BP	GH	VT	SF	RE	MH
**Total sample (N=120)**								
Appraisal of emotions		-	-	-	-	-	-	-	-
Optimism/Regulation		-	-	-	0.31*	0.33*	-	0.30*	0.49***
Utilizing emotions		-	-0.32**	-	-	-0.25*	-0.29*	-0.36**	-0.36**
	Adjusted R^2^	-0.002	0.058	0.004	0.136	0.072	0.040	0.058	0.120
Males (N=80)									
Appraisal of emotions		-	0.50***	-	0.32*	0.29*	0.30*	-	-
Optimism/Regulation		-	-	-	0.30*	-	-	-	0.41**
Utilizing emotions		-	-	-	-	-	-	-	-
	Adjusted R^2^	-0.018	0.136	-0.042	0.241	0.126	0.065	-0.029	0.151
Females (N=40)								
Appraisal of emotions		-	-	-	-	-	-	-	-
Optimism/Regulation		-	0.75*	-	-	-	-	-	-
Utilizing emotions		-	-0.64*	-	-	-0.54*	-0.54*	-0.69**	-0.55*
	Adjusted R^2^	-0.070	0.136	-0.059	0.011	0.098	0.053	0.168	0.093

* *P*<0.05

** *P*<0.01

*** *P*<0.001, according to OLS regressions.

## 4. Discussion

This study aimed at examining quality of life (HRQoL) outcomes and trait emotional intelligence in hospital general managers and to our knowledge it’s the first survey conducted in Greece, which undertakes the aforementioned topic. However, the first results showed that male managers generally exhibited better HRQoL and slightly worse EI compared to their female counterparts, although differences were not statistically significant throughout the sample.

The psychometric properties of the Greek AES were satisfactory. Cronbach’s alpha exceeded the suggested 0.70 threshold for *appraisal of emotions* and *utilization of emotions* and was slightly less (0.645) for *optimism/regulation of emotions*. A possible explanation is that item #28, which hypothetically falls under this factor, also correlates well with *appraisal of* *emotions*, which implies “cross-loading” and that the item may be poorly written (or translated), but may also raise questions about convergent and discriminant validity. Convergent construct validity was supported by the high (>0.40) correlations between items and their hypothesized factors. Discriminant validity was satisfactory as most items correlated remarkably higher with their own, rather than with competing factors.

The significantly higher SF-36 scale scores of this sample, compared to the Greek general population, may have been expected as the hospital managers participating in this study were generally healthy, middle-aged individuals in the upper socioeconomic strata in terms of education, career prospects and income. Education typically affects all health domains, although stronger associations have been reported with mental rather than physical health (Araya et al., 2003). Higher income has also been linked to better health (Papa et al., 2009). The expected gender effect was confirmed as men scored higher on all SF-36 scales, although differences were significant only for *social* *functioning* and *role emotional*. An explanation might be that other health contributors (e.g. education, marital status, income) could be minimizing gender differences in this sample.

Females expectedly scored higher on all EI factors, however differences were significant only for *appraisal of emotions*. This may reflect better judgment and evaluation of own of others’ emotions and in the present study it probably stems from higher awareness of non-verbal messages sent and understanding of feelings from facial expressions and voice tones, as these items demonstrated the most noteworthy gender differences. Other studies have also indicated that EI may vary with gender and particularly that women have somewhat higher scores than men (Goldenberg et al., 2006; Mayer et al., 1999; Schutte et al., 1998; Van Rooy et al., 2005) Contrarily, the within-factor examination of items in the other two EI components showed that only two items (27 and 30) under the *utilization of emotions* factor were scored higher (*p*<0.05) by women. Mean item scores and response frequency distributions are not shown for parsimony but are available from the authors.

No clear trait EI gender differences have been found in some studies (Bar-on, 1997; Bar-on et al., 2000; Brown & Schutte, 2006; Schutte et al., 1998), whereas others have shown mixed results, e.g. women being better at emotional attention and empathy, while men at regulating emotions (Fernadez et al., 2004; Van Rooy et al., 2005). It has been suggested that this lack of uniformity in results could be due to socio-demographic characteristics of the sample and the tool used each time. The latter is linked to the skills comprising the construct, which depend on the theoretical model being dealt with (Sanchez et al., 2004).

These associations observed here between trait EI and HRQoL components (0.18-0.39) are comparable to those between trait EI and other outcomes, e.g. work and academic performance, (Van Rooy & Viswesvaran, 2004) and Big Five Personality Dimensions and symptoms of psychopathology (Malouff et al., 2005*)*. *Appraisal of* *emotions* and *optimism/regulation* correlated with *mental health* and *vitality*. This is consistent with previous reports indicating that higher EI is related to more positive and less depressing mood (Schutte et al., 2002) The theoretical basis for expecting this relationship is that better perception and regulation of emotions helps to better manage fatigue symptoms, develop healthier mood and cope more successfully with stress (Brown & Schutte, 2006).

In male managers, *physical role, general health, vitality* and *social functioning* were predicted by *appraisal of emotions*, whereas *general health* and *mental health* by *optimism/regulation* *of emotions*. *Utilizing emotions* was not a significant predictor of any SF-36 scale in men, but it predicted five scales in women (*physical role, vitality, social functioning, emotional role* and *mental health*), with *role physical* also predicted by *optimism/regulation*. In females, *appraisal of emotions* was not a significant predictor of any SF-36 scale. According to our results, the three trait EI factors explained 6.5%-24.1% and 5.3%-16.8% of the variance in health in men and women respectively, comparable to results from a previous study in which EI had a weighted average association of r=0.29 with mental health, r=0.31 with psychosomatic health and r=0.22 with physical health (Brown & Schutte, 2006).

On the basis of the present and previous studies it may be concluded that gender differences in trait EI cannot be generalized. It should be remembered that trait EI is a range of abilities, self-awareness, emotional self-management, empathy and social skills. Women tend to be better on average at emotional empathy, sensing how the other person is feeling, and at keeping things feeling well between people in a group. Men, on the other hand, tend to be better on average at self-confidence, particularly in group and at managing distressing emotions (Goleman, 2006). Hence, more investigation is required, and the findings of this and other studies should be interpreted cautiously.

This study has some limitations to be considered. As a correlational, cross-sectional study, it is not possible to establish the direction of the relationships. Hence, longitudinal studies are required to determine the causal role of trait EI in the development of symptoms which can affect HRQoL. The EI factors explain 5% to 25% of the variance in HRQoL, which leaves substantial variance unaccounted for. Factors such as stress, burnout, job satisfaction, lifestyle and obviously comorbid illnesses might better account for health, and should be accounted for in future studies. The potential problems raised by the unequal sample size of the gender groups compared should also be taken into consideration (Frazier et al., 2004). Finally, we used only a group of Greek hospital managers to test the relationship between trait EI and health, and this might have implications on the robustness of the results. Hence it is necessary to replicate the results in different samples before attempting any generalizations. In hospital managers, it is also necessary to conduct tests of if (and how) trait EI and its components are actually related to managerial outcomes.

It should be noted that all managers participating in this study were appointed by the MoH through a publicly announced tender, with emphasis on criteria such as education, experience, and producing results. Hence, this study contributes, in a sense, to the emotional and health-related assessment of these managers, while a monthly evaluation of goals, means and results imposed by the MoH also took place for the first time in the Greek NHS, during the same period.

In conclusion, this study clearly raises some issues which can be seen to have both theoretical and practical implications. It provides an indication that there is a positive association between EI and general health, and there appears to be a gender difference in how this occurs. These ideas provide a foundation for further research. From a practical point of view, it might be beneficial to management if practitioners could be trained on individual skills and abilities to promote EI, which might in turn contribute to promoting positive general health. In addition, in future studies it may be worthwhile to add variables that may be amenable to training and interventions that could be useful from an applied perspective.
